# Retrieval and repositioning of an embolized atrial septal defect closure device using a gooseneck snare

**DOI:** 10.1186/s43044-021-00175-4

**Published:** 2021-06-05

**Authors:** Akash Batta, Sanjeev Naganur, Ajay Rajan, Kunwer Abhishek Ary, Atit Gawalkar, Parag Barwad

**Affiliations:** Department of Cardiology, Advanced Cardiac Centre, Post Graduate Institute of Medical Education & Research, Chandigarh, 160012 India

**Keywords:** Atrial septal defect, Transcatheter device closure, Device embolization, Snare

## Abstract

**Background:**

Closure of all haemodynamically significant atrial septal defects (ASDs) is recommended irrespective of symptoms. Percutaneous device closure offers a favourable alternative to surgery with lower morbidity, shorter duration of hospital stays, and avoidance of a surgical scar. Though device closure is generally a safe procedure with high success rates, certain complications can arise including device embolization which poses a significant challenge for the treating team.

We report one such case in which the ASD closure device got spontaneously released and embolized from the delivery cable into the left atrium prior to its deployment. We describe our approach for its retrieval and subsequently its successful deployment across the septal defect using a gooseneck snare.

**Case presentation:**

A 5-year-old asymptomatic child was found to have a murmur on a routine check-up. Evaluation revealed a haemodynamically significant, 18-mm ostium secundum ASD with normal pulmonary pressures and suitable margins for device closure. A 20-mm ASD closure device was traversed via an 8-Fr delivery system. While manipulating the left atrial (LA) disc from the right upper pulmonary vein (RUPV) approach, the device got spontaneously released. The right atrial (RA) disc was caught across the ASD, into the left atrium. This was confirmed by intraoperative transthoracic echocardiography and fluoroscopy. The haemodynamics and rhythm were stable. A 20-mm gooseneck snare was immediately passed through the delivery sheath and an attempt was made to catch the screw. With difficulty, the RA screw was caught with the snare and multiple attempts to retrieve the device into the sheath were unsuccessful. However, while negotiating, we were able to secure a favourable position of the device across the atrial septal defect, and after fluoroscopic and echocardiographic confirmation, the device was released. The child remained stable thereafter and was discharged 2 days later.

**Conclusions:**

Gooseneck snare is a valuable tool in the management of embolized ASD closure device. Occasionally, like in the index case, one may be successful in retrieving the embolized device and repositioning it across the ASD using a gooseneck snare, thus obviating the need for emergency surgery.

## Background

Atrial septal defect (ASD) is a common congenital heart disease (CHD) with a prevalence of 3.2 per 1000 live births and accounts for 7–10% of all CHDs [1]. There are three types of ASDs, namely the ostium primum, ostium secundum and the sinus venosus defects. Ostium secundum defect is the most common type accounting for 75% of all ASDs [2]. Without treatment, haemodynamically significant ASD leads to right ventricular volume overload and eventually failure, atrial tachyarrhythmias, pulmonary hypertension and occasionally paradoxical embolization into the systemic circulation with serious consequences. Mortality rates in these haemodynamically significant defects have been reported to be around 25% in the long run [3]. Hence, closure of all haemodynamically significant ASDs, irrespective of symptoms, is recommended [4]. Traditionally, surgical closure of the defect was considered the gold standard in the management of haemodynamically significant secundum ASD. However, with the advent of percutaneous closure devices, surgery is largely limited to those with larger defects or insufficient rims for device closure. Percutaneous device closure offers a suitable alternative to surgery with lower morbidity, shorter duration of hospital stays and avoidance of a surgical scar [3].

Though device closure is generally a safe procedure with high success rates, certain complications can arise. Device embolization is one such dreaded complication with a reported incidence of 0.5% even in experienced hands [5]. Retrieval of the embolized device poses a significant challenge for the entire treating team and requires discussion and coordination between the interventional cardiologist, cardiac surgeon, interventional imaging specialist, anaesthesiologist and paediatrician. Percutaneous and surgical retrieval options exist.

We report one such case in which the ASD closure device got spontaneously released and embolized from the delivery cable into the left atrium prior to its deployment. We describe our approach for its retrieval and subsequently its successful deployment across the septal defect using a gooseneck snare.

## Case presentation

**Fig. 1:**
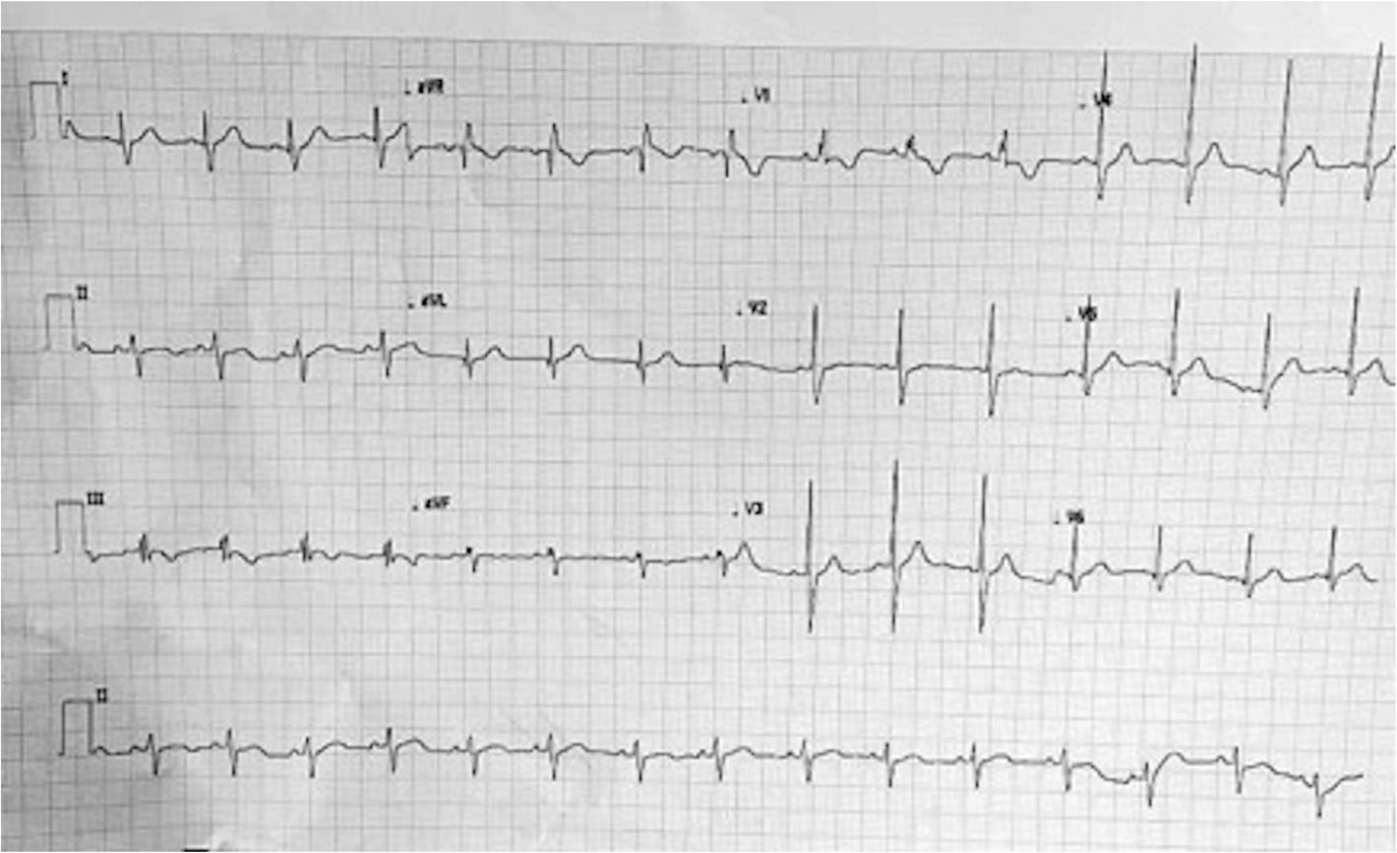
Twelve-lead electrocardiogram showing incomplete right bundle branch block and notching in inferior leads suggestive of crochetage sign seen in secundum atrial septal defects

**Fig. 2:**
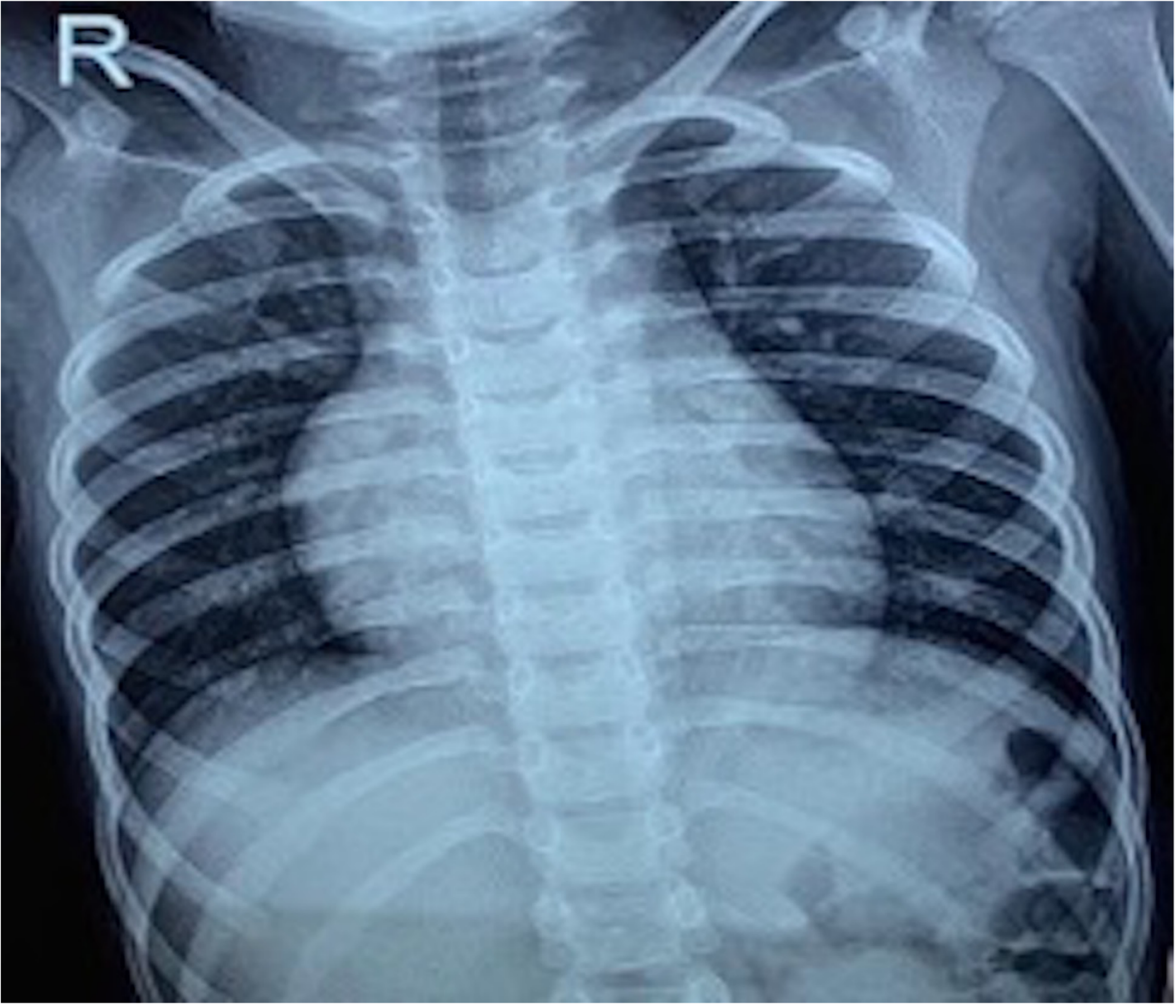
Chest x-ray showing right atrial, pulmonary arterial enlargement with cardiomegaly and right ventricular apex. Numerous end-on vessels are seen over bilateral lung fields suggestive of increased pulmonary blood flow

**Fig. 3:**
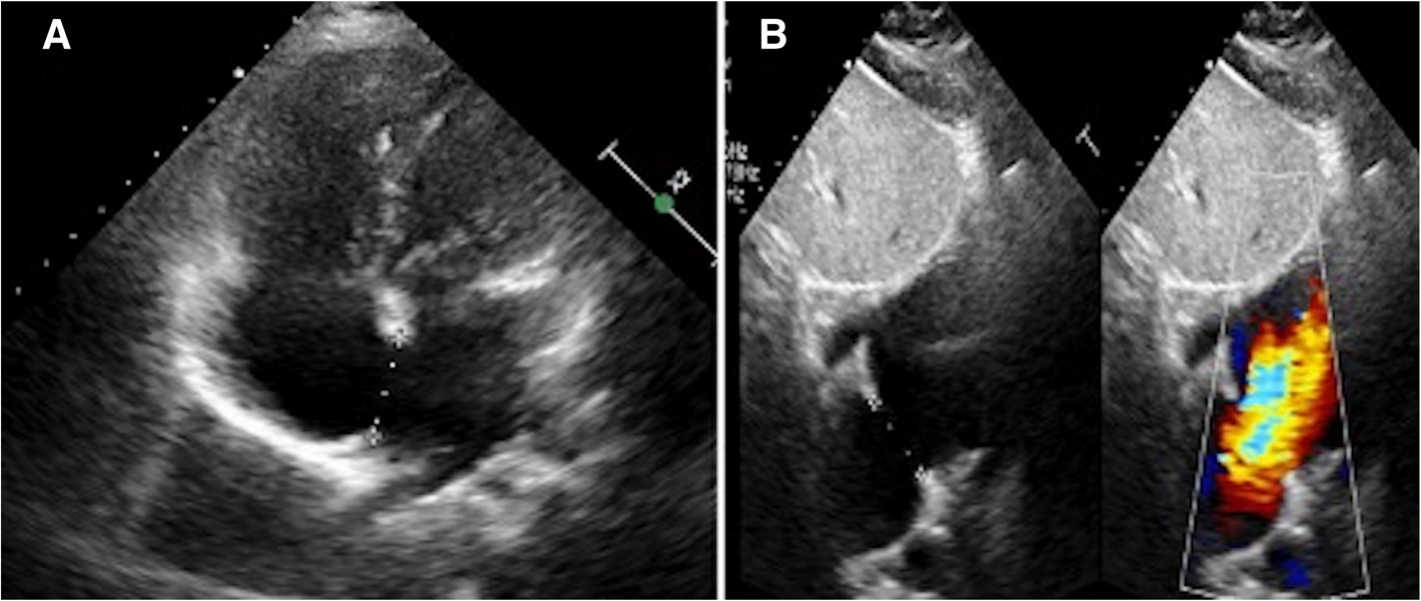
Transthoracic echocardiogram showing an 18-mm Secundum atrial septal defect with dilated Right atrium and right ventricle (**A**) and left-to-right flow across the defect (**B**)

A 5-year-old child was referred by his paediatrician for further evaluation of incidentally detected murmur. He weighed 17 kg with a height of 112 cm. He was doing well otherwise according to his parents. When examined, his saturation was 98% with normal blood pressure (92/60 mmHg) and pulse (90/min). On cardiac examination, the apex was in the 5th intercostal space in the mid-clavicular line. It was diffuse in nature with lateral retraction suggestive of the right ventricular type of apex. The first sound was normal, and the second sound was wide and fixed. There was a grade 2/6 ejection systolic murmur at the pulmonary area without a click. The electrocardiogram (Fig. 1) showed sinus rhythm with rightwards QRS axis and an incomplete right bundle branch block pattern. A chest radiograph (Fig. 2) showed cardiomegaly with increased pulmonary blood flow. A transthoracic echocardiography (TTE) (Fig. 3A, B) showed an 18-mm secundum ASD, shunting left to right with dilated right-sided chambers. There was no pulmonary arterial hypertension. The left chambers were normal with good function. The rims were assessed for suitability for device closure and found to be adequate. We do not use transoesophageal echocardiography on a routine basis for the assessment of suitability for device closure. Hence, the child was planned for an elective percutaneous closure of the defect with a 20-mm ASD device.

**Fig. 4:**
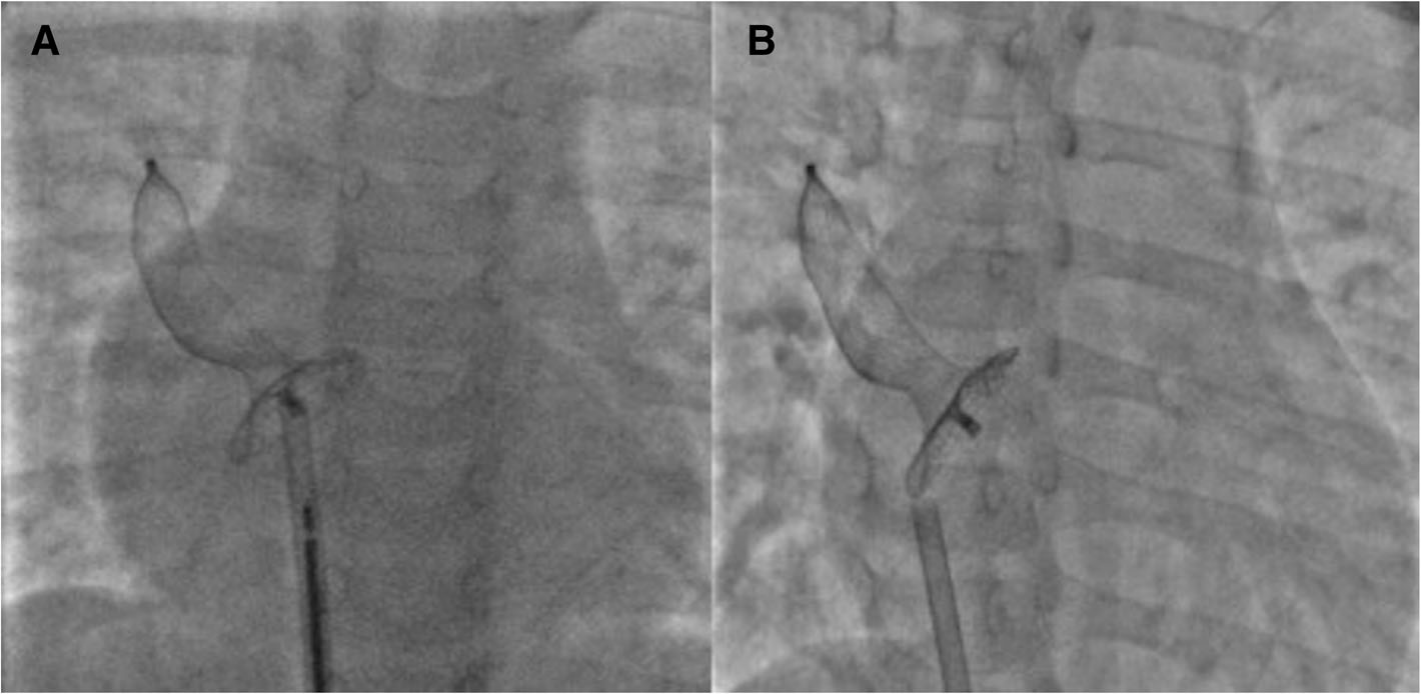
Spontaneous detachment of the cable from the ASD closure device while manipulating from the right upper pulmonary vein (**A**) followed by loss of alignment (**B**)

**Fig. 5:**
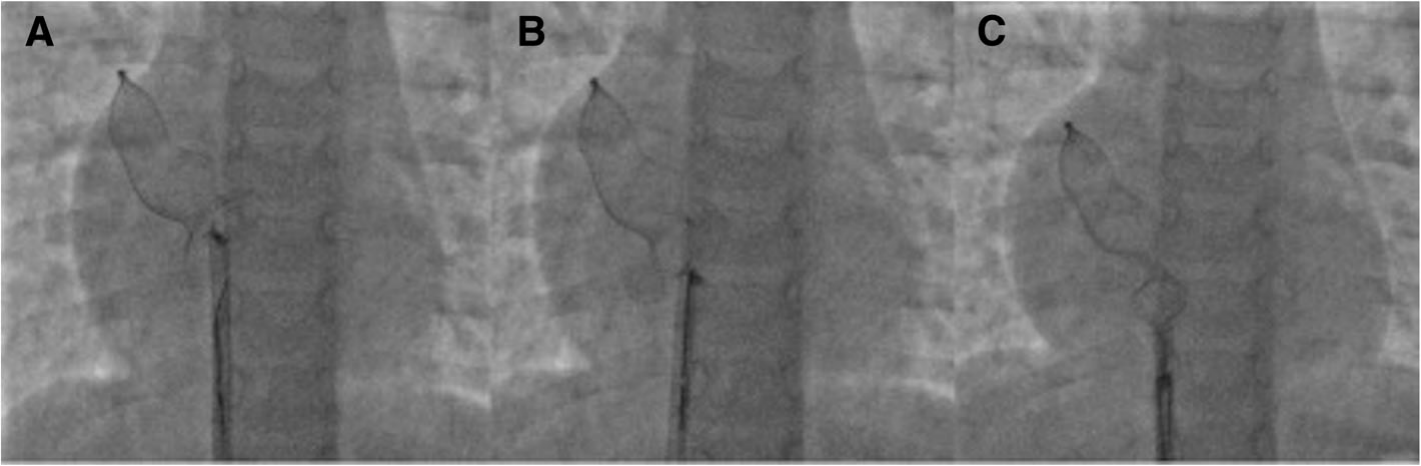
Advancement of a 20-mm gooseneck snare over a larger sheath (**A**). Capture of screw on right atrial disc (**B**) and subsequent attempts to retrieve the device back into the sheath (**C**). The device however could not be retrieved into the sheath

**Fig. 6:**
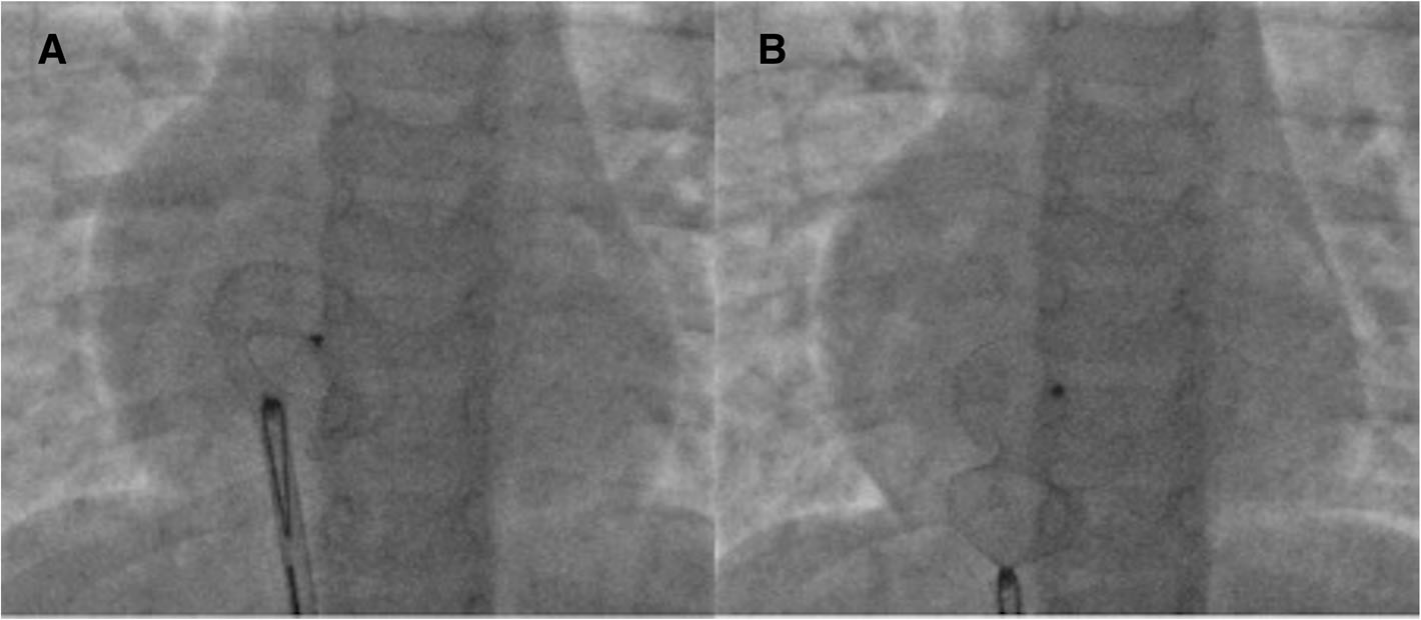
Manipulation of the device with a snare passed over the delivery sheath. The nicks in the delivery sheath could have helped in securing alignment across the septal defect (**A**). Controlled Minnesota manoeuvre with the snare (**B**)

**Fig. 7:**
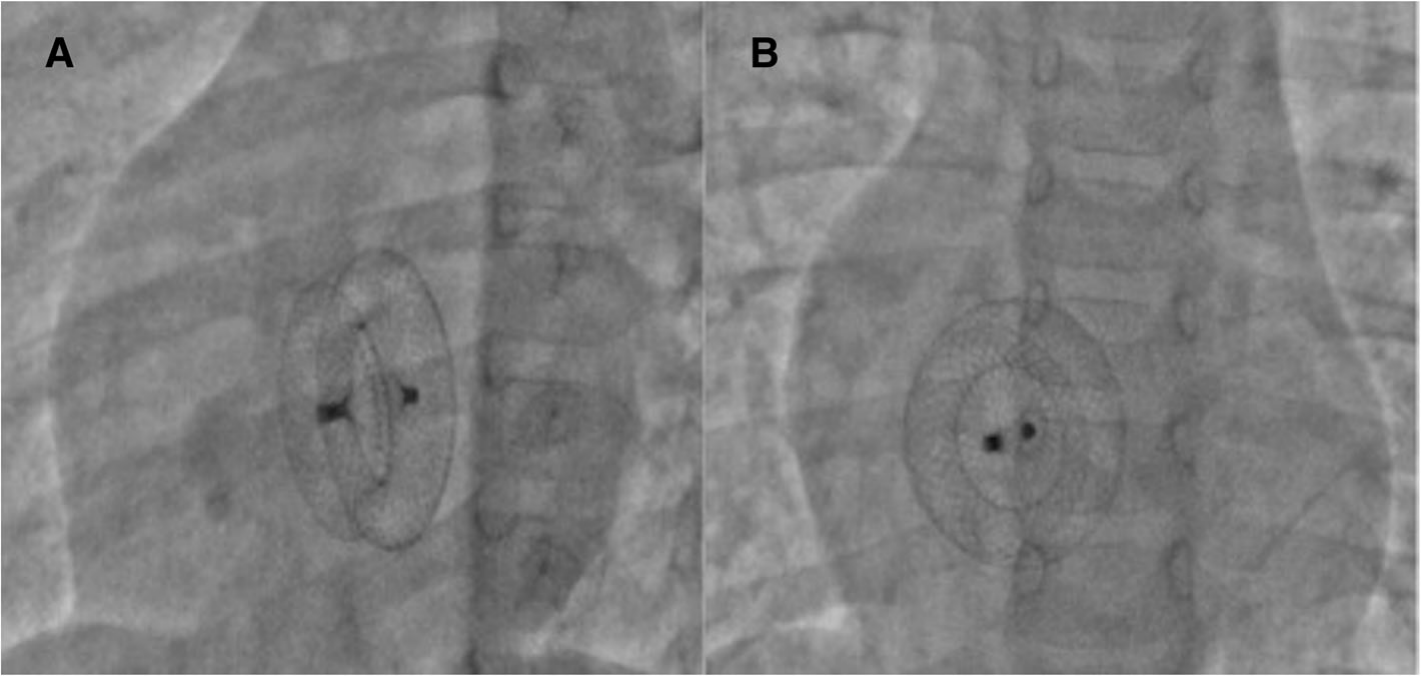
Final fluoroscopy images after the release of ASD closure device from the snare in left anterior oblique 45° (**A**) and anteroposterior views (**B**) confirming the proper positioning of the device across the defect

**Fig. 8:**
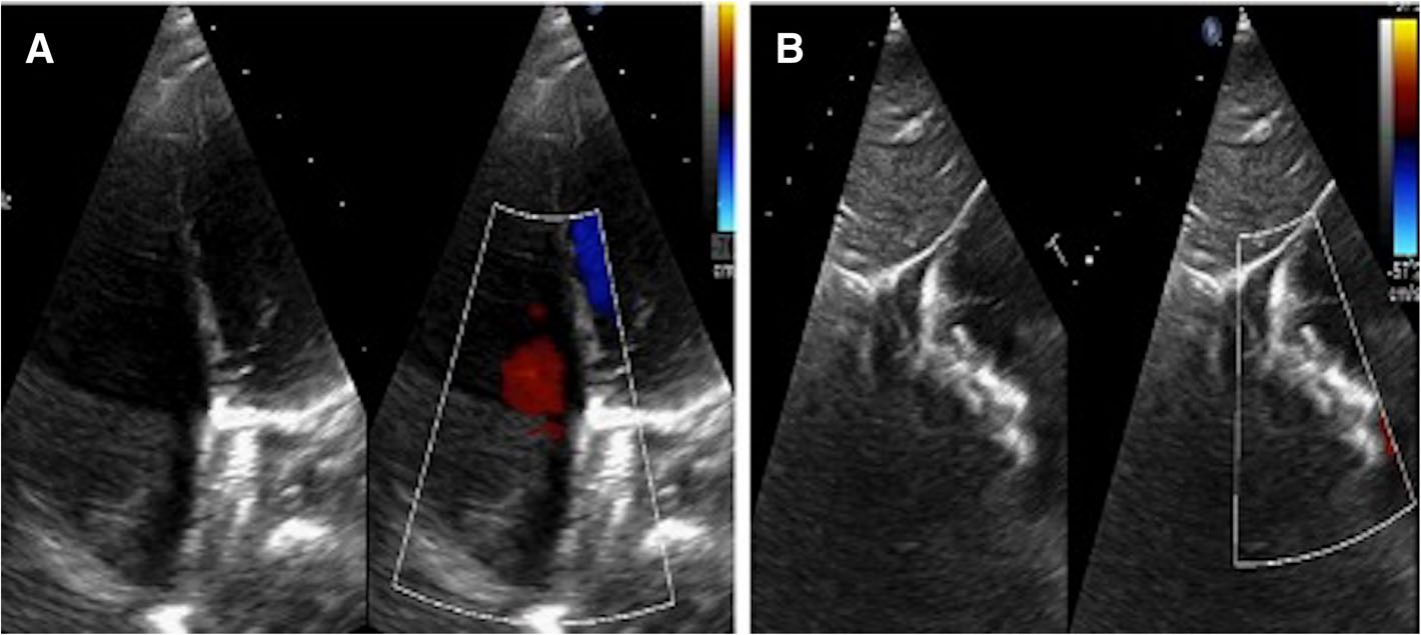
Final transthoracic echocardiographic images confirming the proper positioning of the device across the interatrial septum in apical 4-chamber (**A**) and subcostal (**B**) views

As per our unit’s policy, after written informed consent from both parents, catheterization and the device closure of ASD was performed under intravenous sedation. We do not use transoesophageal echocardiography on a routine basis for device closure of ASDs. The standard digital palpation was used to establish the right femoral vein. The pulmonary artery pressure was 30/12 (mean 18) mmHg, with Qp/Qs of 2.1:1. ASD was crossed with a 6-Fr multipurpose diagnostic catheter using a Terumo glidewire and placed in the right upper pulmonary vein (RUPV) (Fig. 4A) A 20-mm ASD closure device (Cocoon septal occluder, Vascular Innovations Co. Nonthaburi, Thailand) was traversed via an 8-Fr delivery system. While manipulating the left atrial (LA) disc from the RUPV approach, the device got spontaneously released with the right atrial (RA) disc caught across the ASD (Fig. 4B). It was further confirmed by intraoperative transthoracic echocardiography and fluoroscopy. The device position was found to be stable although not in the right place. The haemodynamics and rhythm status were stable. A 20-mm snare (Amplatz GooseNeck, single-loop Snare, eV3 Endovascular, Inc., Part of Covidien; Plymouth, MN, USA) was immediately passed through the delivery sheath and an attempt was made to catch the screw. It was difficult to pull the caught screw into the sheath as the device was unusually aligned (Fig. 5A–C). As upsizing of the sheath was also not helpful, we made a few nicks at the tip of the sheath making its end wider (Fig. 6A), which could have allowed the device to get aligned with the lumen of the sheath. This technique helped us catch and manipulate the device in a favourable manner. The result was the release of the stuck LA disc from RUPV and proper alignment of the device across the defect. After conforming the device position in different (anteroposterior and left anterior oblique 45°) fluoroscopic views, and on transthoracic echocardiography, we performed Minnesota wiggle to ensure the stability of the device position (Fig. 6B). The device was released under fluoroscopic and transthoracic echocardiographic guidance (Fig. 7A, B), with post release echocardiography showing a stable position of the device with no residual shunt (Fig. 8A, B). There were no further complications, and the child withstood the entire procedure well. The total procedure time was 45 min with the fluoroscopic time of 10 min. Haemostasis was achieved with digital compression and was monitored in the intensive care unit for 12 h. Low molecular weight heparin was administered for 2 days, and he was started on aspirin at 3 mg/kg/day once daily. The child was discharged on day 3 of the procedure.

## Discussion

The indications for closure of an ASD include any haemodynamically significant shunt with an increased pulmonary blood flow (Qp/Qs of > 1.5:1), transient right to left shunt leading to transient ischaemic attacks, stroke or cyanosis. In general, transcatheter closure is preferred; however, for defects larger than 38 mm or lacking sufficient rims for supporting the septal occluder devices, surgical closure is recommended [4]. Secundum ASD remains the primary defect amenable for device closure. Studies have shown that TTE provides comparable information to transesophageal echocardiography and suffices in most cases when an adequate echocardiographic window is available. Further, it may lead to decreased procedural time, the need for general anaesthesia and its attendant complications [6].

Success rates of transcatheter device closure are reported to be around 98% and residual shunting after deployment is uncommon in the long term [7]. Major complications of device closure include device embolization, erosion, atrial arrhythmias, pericardial effusion, atrioventricular block and thromboembolism [7]. Though the incidence of device embolization has gone down in recent years, it remains a serious complication. Various factors that predispose to device embolization include larger defect size with floppy or deficient rims, thin atrial septal tissue, type of the device used, use of undersized device and change in position of the device after its deployment. Excessive tension on the delivery cable or excessive wiggle manoeuvring may increase the chances of embolization. Device embolization can occur at any time ranging from immediately post deployment on the table to several weeks and months later [7].

Around 50–75% of all embolized devices can be retrieved successfully with percutaneous methods, and surgery may be necessary in others [8]. Early device embolization is largely restricted to LA. However, embolization to the right ventricle, pulmonary artery and systemic circulation have all been reported [7]. Retrieval via percutaneous methods generally demands the stability of the device first, which may be accomplished by using a stiff guidewire or a bioptome [9]. The next step is to use the snare to catch the screw on the RA disc and subsequently to try and pull the device into the sheath. This step is crucial and necessitates proper force application at an appropriate degree of alignment of the device with the sheath. It is advisable to use a 2-Fr larger and stiffer sheath, to facilitate the easy entry of the RA disc. Retrieval via snaring the LA disc screw should be avoided [5].

Failure of the percutaneous retrieval methods may necessitate a surgical approach especially when the device embolizes outside the heart. Surgery poses a higher risk given the emergent nature of the procedure in most cases, and hence, all attempts should be made to retrieve the embolized device percutaneously.

In the index case, we were successful not only in retrieving the embolized ASD closure device using a gooseneck snare, but also in its successful repositioning with the use of a snare without pulling the device out of the body.

## Learning points

1. Device embolization is an uncommon but serious complication during transcatheter ASD device closure
2. Percutaneous retrieval of embolized ASD closure device is possible in the majority of cases and is a safe and preferred option of device retrieval
3. Use of gooseneck snare to catch the screw on RA disc of ASD closure device can help not only in device retrieval but can also guide its successful repositioning across the ASD

## Conclusions

Though transcatheter device closure of ASD is a relatively safe procedure with a high technical success, complications including device embolization can arise. Retrieval is best performed via percutaneous methods which obviates the need for emergency surgery and its attendant risks. The use of a gooseneck snare is often necessary for these situations. Occasionally, like in our index case, one may successfully retrieve and reposition the device across the defect using the snare.

## Data Availability

The datasets used and/or analysed during the current study are available from the corresponding author on reasonable request

## Abbreviations

ASD: Atrial septal defect
CHD: Congenital heart disease
RUPV: Right upper pulmonary vein
LA: Left atrium
RA: Right atrium
TTE: Transthoracic echocardiography
